# Multidirectional Image Sensing for Microscopy Based on a Rotatable Robot

**DOI:** 10.3390/s151229872

**Published:** 2015-12-15

**Authors:** Yajing Shen, Wenfeng Wan, Lijun Zhang, Li Yong, Haojian Lu, Weili Ding

**Affiliations:** 1 Mechanical and Biomedical Engineering Department, City University of Hong Kong, Tat Chee Avenue, Kowloon, Hong Kong, China; wfwan2-c@my.cityu.edu.hk (W.W.); lzhan23@cityu.edu.hk (L.Z.); haojianlu2-c@my.cityu.edu.hk (H.L.); 2 CityU Shenzhen Research Institute, Shen Zhen, 8 Yuexing 1st Road, Shenzhen 518000, China; 3 Institute of Electrical Engineering, Yanshan University, 438 Hebei Street West Section, Haigang, Qinhuangdao 066004, China; leoqiulin@126.com; 4 College of Electromechanical Engineering, University of Petroleum (East China), No. 66, Changjiang West Road, Huangdao District, Qingdao 266580, China

**Keywords:** multidirectional imaging, robot, microscopy image sensing, micromanipulation

## Abstract

Image sensing at a small scale is essentially important in many fields, including microsample observation, defect inspection, material characterization and so on. However, nowadays, multi-directional micro object imaging is still very challenging due to the limited field of view (FOV) of microscopes. This paper reports a novel approach for multi-directional image sensing in microscopes by developing a rotatable robot. First, a robot with endless rotation ability is designed and integrated with the microscope. Then, the micro object is aligned to the rotation axis of the robot automatically based on the proposed forward-backward alignment strategy. After that, multi-directional images of the sample can be obtained by rotating the robot within one revolution under the microscope. To demonstrate the versatility of this approach, we view various types of micro samples from multiple directions in both optical microscopy and scanning electron microscopy, and panoramic images of the samples are processed as well. The proposed method paves a new way for the microscopy image sensing, and we believe it could have significant impact in many fields, especially for sample detection, manipulation and characterization at a small scale.

## 1. Introduction

Image sensing at a small scale is essential in many fields, such as for the MEMS device defect detection [1,2], precise manipulation [3,4,5], micromaterial characterization [6,7] and so on. In these tasks, a microscope is usually required to enlarge the micro object to observe the details clearly. Here, a significant challenge lies in the field of view (FOV) of the microscope, which would become very small as the microscope’s magnification increases. Therefore, approaches for extending the viewing area of the object at high magnification are attracting increasing interest nowadays.

To extend the FOV of the microscope and to get more image information, some novel microscopes have recently been developed, including the confocal microscope, dual-beam electron microscope (EM), cryo-electron microscope and so on. For instance, confocal microscopy is able to scan half-transparent objects layer-by-layer and then construct a 3D image [8,9]; the dual-beam EM can provide 3D image information by an etching-scanning process [10,11]; the cryo-EM allows one to construct the 3D structures of protein at atom scale [12,13]. Although these techniques have been successfully used in some specific fields, when it comes to multidirectional image sensing, these methods either destroy the samples permanently or have special sample preprocessing requirements.

Another effective approach to extend the sensing FOV is to employ a moveable sample stage for microscopy. Compared with developing new microscopy techniques, this approach is more acceptable and practicable for general applications since the stage can easily be integrated with most of the current microscopies. For instance, a moveable x-y sample stage is able to extend the imaging area of the sample greatly, and an image mosaic can be achieved by standard image processing [14].To view the non-top region of the sample, the tilting stage has also recently been proposed for microscopy, including both optical and electron microscopy. Compared with the x-y stage, the tilting stage is able to tilt the sample to a certain degree under the microscope, whereby it possible to image the sample from different orientations and to construct a 3D structure. For instance, this technique has been successfully used to observe biological cells under an optical microscope (OM) [15,16], and fracture surfaces with subsequent 3D reconstruction under a scanning electron microscope (SEM) [17,18], and nanomaterials under a transmission electron microscope (TEM) [19,20]. However, in this method, more than half of the sample data still cannot be obtained due to the small tilting angles possible. It is obvious that the local properties of the sample cannot fully reflect the bulk properties of the whole object. More seriously, the resulting inadequate image sensing information sometimes may lead to misleading results. For instance, sample defects perhaps can’t be detected if they are located outside the viewing area. Although some sample holders and manipulators allow 360° rotations [21,22,23], they are either unable to always keep the points of interest in focus or it is relatively difficult to assemble samples. Therefore, truly multi-directional image sensing is urgently required at the current stage of the technology. The availability of rich image information from different orientations could benefit the deep understanding of the sample, and promote the study of defect detection, material characterization and other related fields. 

With the rapid progress of robotic techniques, robots have come to be regarded as powerful systems for research at a small scale [24,25,26,27]. For instance, they have been successfully applied for precise manipulation, *in-situ* characterization, and so on, but little literature has reported the use of a robot for image sensing at a small scale, and even less so for imaging micro samples from multiple directions.

Herein, this paper proposes a novel multidirectional image sensing approach for micro object observation under a microscope based on a robot. First, a robot system with endless rotation ability is developed and integrated with a microscope. Then, the micro object is aligned to the rotation axis of the robot automatically based on the proposed alignment strategy. After that, images of the sample are taken from multiple directions by rotating the robot within one revolution under the microscope, and panoramic images of the sample are processed as well. Lastly, we test various types of micro samples under both optical and scanning electron microscopes to demonstrate the versatility of this approach.

## 2. Experimental Section

### 2.1. Robotic System

**Figure 1:**
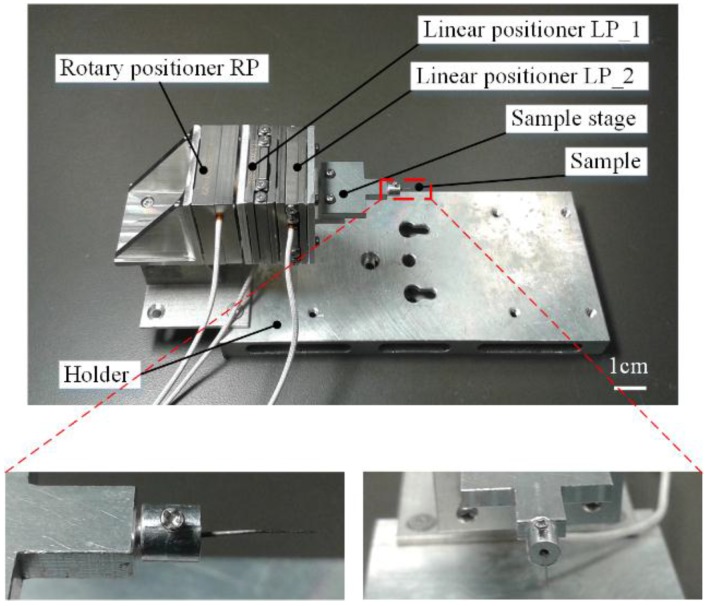
The nanorobotic system for the multidirectional image sensing at small scale, and zoomed picture for the sample holder. The sample holder consists of a hole to hold sample and a screw to fasten the sample.

The designed robot for multidirectional image sensing at a small scale is illustrated in Figure 1. The robot is mainly constructed from three nano-positioners: two linear positioners (ECS3030, Attocube Inc., Muenchen, Germany) and one rotary positioners (ECR3030, Attocube Inc., Muenchen, Germany). The first linear positioner (LP_1) is mounted on the rotary positioner (RP) and the second linear positioner (LP_2) is mounted on the first linear positioner LP_1. LP_1 and LP_2’s movement directions are mutually perpendicular. Each nano-positioner of the robot is responsible for one independent movement, thereby the robot has three degrees of freedom (DOFs) in total: two mutually perpendicular translational movements and one rotation. Additionally, we also designed a T-shape sample stage to hold the sample, a connector to connect RP and LP_1, and a holder to support the whole robot. The sample holder consists of a hole to hold a sample and a screw to fasten the sample.

The travel range, resolution and repeatability of the two linear positioners LP_1 and LP_2 are 20 mm, 1 nm and 50 nm, respectively. The travel range, resolution and repeatability for the rotary positioner RP are 360° endless, (1 × 10^−6^)° and 5% over the full range, respectively. Therefore, the positioning accuracy of the robot can be guaranteed during the image sensing process. 

### 2.2. Sample Alignment

**Figure 2:**
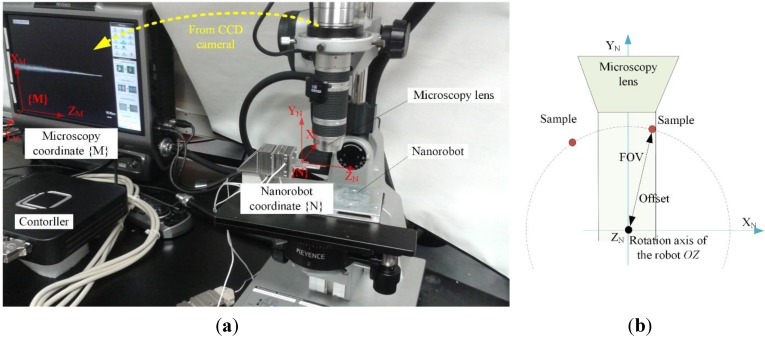
Illustration of the system setup for sample alignment and imaging. (**a**) Two coordinates {M} and {N} are established to represent the microscope coordinates and the robot coordinates and (**b**) shows that the sample may move out of the FOV before it is aligned to the rotation axis of the robot.

During the experiment, the to-be-imaged micro object is assembled on the sample stage of the robotic system, which is put underneath the lens of the microscope, as shown in Figure 2a. To image the sample from multiple directions, the sample should be able to rotate endlessly over 360°. However, one serious problem is that the field of view (FOV) of the microscope is limited, which means the sample may move out of the FOV during the rotation, as illustrated in Figure 2b. To avoid this, sample alignment must be considered before imaging.

Unlike the traditional macro scale alignment, the microscope can only provide 2D image information. Because the imaging direction is perpendicular to the rotation axis, it’s very difficult to obtain the positon of the sample directly based on the microscope image. To address these challenges, we propose an automatic forward-backward alignment strategy. 

As illustrated in Figure 2a, two coordinate systems are established first, *i.e.*, the microscopy coordinates {M} and the robot coordinates {N}. The microscope image coordinates {M} are established on the imaging plane of the microscope with the origin being the lower left corner of the microscope images. The X_M_ axis and Z_M_ axis are along with microscope images’ two sides, and the Y_M_ axis is parallel to the OM’s optical axis. The robot coordinates {N} are established on the robot with the origin being on the rotation axis of RP and also on the outer surface of LP_2. The directions of the X_N_ axis and Y_N_ axis are parallel with the movement directions of LP_2 and LP_1 respectively. As RP rotates, the X_N_ axis and Y_N_ axis would also rotate.

**Figure 3:**
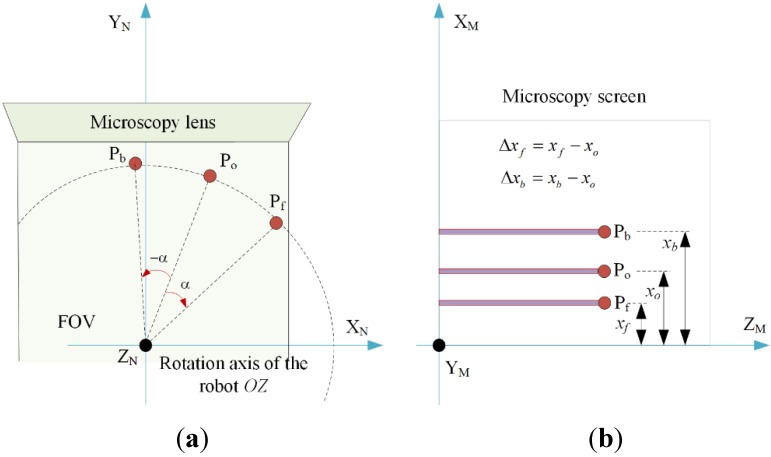
Illustration of the forward-backward alignment approach. (**a**) The position of point P in {N} at the initial position, after forward rotation (clockwise) and backward rotation (anticlockwise); (**b**) The coordinates of point P in {M} during the alignment procedure.

Given a to-be-aligned point P on the sample with initial coordinates in {N}. If we rotate the robot by an angle clockwise and anticlockwise, respectively, the coordinates of point P in {M} before rotation (Figure 3a), after rotating a certain number of degrees clockwise forward and anticlockwise backward can be expressed by Equation (1) [28]:

(1) $$\left[\begin{array}{l} x_{i}\\ y_{i}\\ z_{i} \end{array}\right]=\frac{1}{u}RX(\theta_{x0})\cdot RY(\theta_{y0})\cdot RZ(\theta_{z0}+n_{i})\left[\begin{array}{c} x_{n0}\\ y_{n0}\\ z_{n0} \end{array}\right]+T_{MN}$$

where i=o,f,b, represent point P in {M} before rotation (Figure 3a), after rotating clockwise forward and after rotating anticlockwise backward, respectively. $n_{o}=0$, $n_{f}=\alpha$, $n_{b}=-\alpha$, *u* is the represented physical distance (µm) of each pixel in the microscope image; RX, RY and RZ represent the rotation matrix of {N} relative to {M} about the X_M_-axis, Y_M_-axis, and Z_M_-axis, respectively; T_MN_ is the translational matrix between the two coordinate systems. We define $\Delta x_{f}$ and $\Delta x_{b}$ (Figure 3b) which represents the position shift of point P on the microscope images after rotating clockwise and rotating anticlockwise, respectively (i=f,b):

(2) $$\left[\begin{array}{c} \Delta x_{i}\\ \Delta y_{i}\\ \Delta z_{i} \end{array}\right]=\left[\begin{array}{c} x_{i}-x_{o}\\ y_{i}-y_{o}\\ z_{i}-z_{o} \end{array}\right]=\frac{1}{u}RX(\theta_{x0})\cdot RY(\theta_{y0})\cdot [RZ(\theta_{z0}+n_{i})-RZ(\theta_{z0})]\left[\begin{array}{c} x_{n0}\\ y_{n0}\\ z_{n0} \end{array}\right]$$

Angles $\theta_{x0}$, $\theta_{y0}$, and $\theta_{z0}$ represent the initial rotation angle of {N} relative to {M} about the X_M_ axis, Y_M_ axis and Z_M_ axis, respectively. The three angles ($\theta_{x0}$, $\theta_{y0}$, $\theta_{z0}$) are manually compensated to be zero before the automatic alignment experiments. Angle $\theta_{x0}$ is compensated to be zero by adjusting the Y_N_ axis to be parallel with the Y_M_ axis. Angle $\theta_{y0}$ is compensated to be zero by adjusting the robot’s holder so that robot’s edges are parallel with the image plane’s edges. Angle $\theta_{z0}$ is compensated to be zero by rotating the rotary positioner to make the X_M_ axis parallel with the X_N_ axis.

Then, Equations (4) and (5) can be simplified to:

(3) $$\left[\begin{array}{c} \Delta x_{f}\\ \Delta y_{f}\\ \Delta z_{f} \end{array}\right]=\frac{1}{u}[RZ(\alpha )-RZ(0)]\left[\begin{array}{c} x_{n0}\\ y_{n0}\\ z_{n0} \end{array}\right]=\frac{1}{u}\left[\begin{array}{ccc} \cos\alpha -1 & \sin\alpha & 0\\ -\sin\alpha & \cos\alpha -1 & 0\\ 0 & 0 & 0 \end{array}\right]\left[\begin{array}{c} x_{n0}\\ y_{n0}\\ z_{n0} \end{array}\right]$$

(4) $$\left[\begin{array}{c} \Delta x_{b}\\ \Delta y_{b}\\ \Delta z_{b} \end{array}\right]=\frac{1}{u}[RZ(-\alpha )-RZ(0)]\left[\begin{array}{c} x_{n0}\\ y_{n0}\\ z_{n0} \end{array}\right]=\frac{1}{u}\left[\begin{array}{ccc} \cos\alpha -1 & -\sin\alpha & 0\\ \sin\alpha & \cos\alpha -1 & 0\\ 0 & 0 & 0 \end{array}\right]\left[\begin{array}{c} x_{n0}\\ y_{n0}\\ z_{n0} \end{array}\right]$$

By solving the above two Equations (6) and (7), the to-be-aligned point P’s coordinate ${\left(x_{n0},y_{n0},z_{n0}\right)}^{T}$ in {N} can be expressed by:

(5) $$\left[\begin{array}{c} x_{n0}\\ y_{n0}\\ z_{n0} \end{array}\right]=u\left[\begin{array}{c} \frac{\Delta x_{f}+\Delta x_{b}}{2\cos\alpha -2}\\ \begin{array}{l} \frac{\Delta x_{f}-\Delta x_{b}}{2\sin\alpha }\\ \frac{1}{u}z_{n0} \end{array} \end{array}\right]$$

In Equation (8), $\Delta x_{f}$ and $\Delta x_{b}$ can be measured from the microscope by image processing; α is the rotation angle defined by the users; µ (um/pixel) represents the physical distance of each pixel, which can be calibrated based on the microscope’s magnification. After the to-be-aligned P’s coordinates ${\left(x_{n0},y_{n0}\right)}^{T}$ in {N} are obtained, the sample can be aligned to the rotation axis of RP by moving LP_1 by $-y_{n0}$ and LP_2 by $-x_{n0}$, respectively.

The proposed alignment approach is based on the “to-be-aligned point”. Therefore, it can theoretically successfully align the “point of interest” to the rotation axis of the robot regardless of the installation position of the sample. However, in real practice, we always expect to view more regions in a single image. In this case, the sample needs to be aligned carefully to make most of the surfaces have a similar focus depth during the movement. Thus, in this paper, a T-shape sample stage is developed to hold the sample in a direction vertical to the microscope lens, by which a wide region of the sample can be imaged clearly during the rotation. 

Finally, to verify the viability and robustness of this alignment approach, we implement a sample alignment experiment based on the method proposed above (see the Supplementary Material for details). The results prove that this alignment approach has high repeatability regardless of the rotation speed, and the alignment accuracy can meet the requirement for multi-directional imaging under a microscope.

### 2.3. Multidirectional Imaging

After the sample is aligned to the rotation axis of the robot, the sample is able to remain in the FOV of the microscope during the rotation. Thus, a series of images can be taken from different directions by continuously rotating the robot. 

In this experiment, the travel speed of the linear positioner and the rotary positioner are set to 667 µm/s and 5 °/s, respectively; the rotation angle α is chosen as 5°. After the sample is aligned to the rotation axis of the robot, the focus of the microscope should be manually adjusted once to focus on the sample. After that, we can rotate the robot endlessly to view the sample from multiple directions freely, during which there is no need to adjust the focus any more.

**Figure 4:**
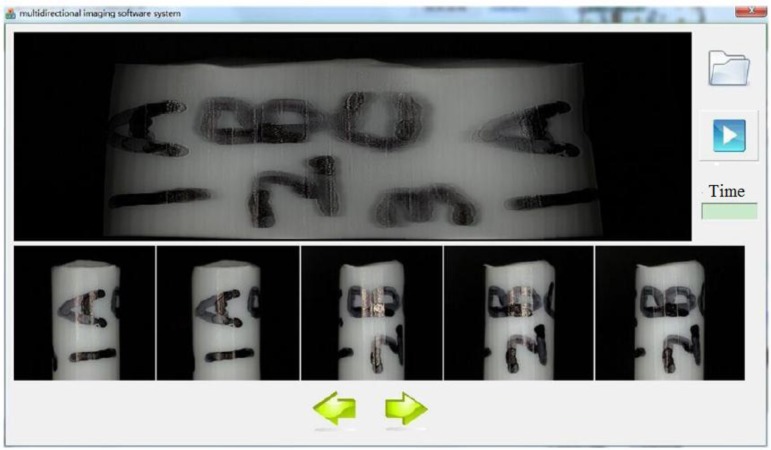
Developed software system to create panoramic image from a series of images taken from different rotation angles.

Next, to further illustrate the image information that can be obtained, we reconstructed a panoramic image by automatic panoramic image stitching. We developed a new multidirectional imaging software system, which combines a serial of input images (the small images at the bottom of the Figure 4) into a panoramic image (the image at the top of the Figure 4), and creates a new projection of the panoramic image. The core contribution of the software is the new designed image stitching algorithm according to the characteristics of the robotic system. The stitching process can be divided to three steps [29], *i.e.*, feature matching, image matching, and bundle adjustment.

#### 2.3.1. Feature Matching

The first step in our algorithm is to extract and match scale-invariant feature transform (SIFT) features between all of the images. SIFT features are located by using maxima and minima of the result of different Gaussian functions applied in scale space to a series of smoothed and re-sampled images. In our system, the SIFT features are extracted directly by using functions for detecting SIFT image features, which are written based on [30]. Here, low contrast candidate points and edge response points along an edge are discarded, and dominant orientations are assigned to localized SIFT features. These steps ensure that the SIFT features are robust to changes in illumination, noise, and minor changes in viewpoint. In addition to these properties, they are highly distinctive, relatively easy to extract and allow for correct object identification with low probability of mismatch. Thus, our algorithm is more stable for matching and can handle images with varying illumination and noise. Once features have been extracted from all n images, robust correspondences are required in order to estimate the necessary transformation to align an image with the next image.

In our robotic system, the robot rotates the axis of OZ, and a series of images of the sample can be taken continuously with a fixed rotation angle α. Under this condition, only neighboring images are searched for matching features, and the distances between all matching features in the neighboring images have the similar values of z. According to these characteristics, a new constraint condition is added to help the feature matching stage. 

Assuming that any SIFT features detected in the current image is defined as $u_{o}=[x_{mo},z_{mo}]$, we search the matching SIFT feature in the next image by using a k-d tree algorithm [31], which finds the nearest neighbour of the given input point in the tree. Here, the searching range of k-d tree is limited as $x_{mf}\in [0,width],z_{mf}\in [z_{mf}-\sigma ,z_{mf}+\sigma ]$, and the result is define as $u_{f}=[x_{mf},z_{mf}]$. In this way, the result of the search is more accurate and execution of the comparison is faster.

#### 2.3.2. Image Matching

Assuming that the robot rotates the axis of RP, which is equivalent to the microscope rotates about its optical center, the group of transformations the images may undergo is a special group of homographies. We denote each robot’s location as 3 rotation angles $\text{α}=\left[\alpha_{1},\alpha_{2},\alpha_{3}\right]$ and the work distance of the microscope as f. For small changes in image position, this gives pairwise homographies:

(6) $$u_{o}=H_{of}u_{f}=K_{o}R_{o}{R_{f}}^{T}{K_{f}}^{-1}u_{f}$$

where $H_{of}=K_{o}R_{o}{R_{f}}^{T}{K_{f}}^{-1}$, $u_{o}=[x_{mo},z_{mo}]$, $u_{f}=[x_{mf},z_{mf}]$, $K_{o}=\left[\begin{array}{ccc} f_{o} & 0 & 0\\ 0 & f_{o} & 0\\ 0 & 0 & 1 \end{array}\right]$$R_{o}=e^{\left[\alpha_{o}\right]},\;[\alpha_{o}]=\left[\begin{array}{ccc} 0 & -\alpha_{o3} & \alpha_{o2}\\ \alpha_{o3} & 0 & -\alpha_{o1}\\ -\alpha_{o2} & \alpha_{o1} & 0 \end{array}\right]$

After feature matching, the homography can be calculated by the matches between the images. For each image, we consider m candidate matching images, which have the greatest number of feature matches to this image. Then we find geometrically consistent feature matches using RANSAC algorithm [32] to estimate the homography. Here, we select sets of r=4 feature correspondences and compute the homography $H_{of}$ between them using the direct linear transformation (DLT) method [33]. And we repeat this with n = 500 trials and find the correct solution that has the maximum number of inliers. Furthermore, to obtain multi-directional imaging with a set of images and reject noise images which match to no other images, the probabilistic model proposed in [29] is also applied to verify the match. 

#### 2.3.3. Bundle Adjustment

For each connected component, bundle adjustment [34] is performed to solve for the rotation $\alpha_{1}$, $\alpha_{2}$, $\alpha_{3}$ and the working distance f of the microscope. That is, each feature is projected into all the images in which it matches, and the sum of squared projection error is minimized with respect to the camera parameters. Given a correspondence $u_{o}^{k}↔u_{f}^{l}$, $u_{o}^{k}$ denotes the position of the *k*th feature in image *o*, the residual is:

(7) $${r_{ij}}^{k}={u_{i}}^{k}-K_{o}R_{o}{R_{f}}^{T}{K_{f}}^{-1}u_{f}$$

In order to get the parameters of the microscope, the sum of the squared projection error is defined as follows:

(8) $$e=\left\{ \begin{array}{l} \sum_{o=1}^{n}\sum_{f\in \kappa (o)}\sum_{k\in F(o,f)}{\left|r_{of}\right|}^{2},if\left|r_{of}\right|<\sigma \\ \sum_{o=1}^{n}\sum_{f\in \kappa (o)}\sum_{k\in F(o,f)}2\sigma \left|r_{of}\right|-\sigma^{2},if\left|r_{of}\right|\geq \sigma \end{array}\right.$$

where σ is a distance threshold. We use an outlier distance σ=∞ during initialization and σ=2 pixels for the final solution. Then the parameters are updated using Levenberg-Marquard algorithm. Each iteration step is of the form:

(9) $$\Phi ={\left(J^{T}J+\lambda {C_{p}}^{-1}\right)}^{-1}J^{T}r$$

where Φ are all the parameters, r is the residual and. We $J=\frac{\partial r}{\partial \Phi }$ encode our prior belief about the parameter changes in the covariance matrix $C_{p}$:

(10) $$C_{p}=\left[\begin{array}{cccccc} {\sigma_{\alpha }}^{2} & 0 & 0 & 0 & 0 & \cdots \\ 0 & {\sigma_{\alpha }}^{2} & 0 & 0 & 0 & \cdots \\ 0 & 0 & {\sigma_{\alpha }}^{2} & 0 & 0 & \cdots \\ 0 & 0 & 0 & {\sigma_{f}}^{2} & 0 & \cdots \\ 0 & 0 & 0 & 0 & {\sigma_{\alpha }}^{2} & \cdots \\ \vdots & \vdots & \vdots & \vdots & \vdots & \ddots \end{array}\right]$$

Considering the rotate angle of the robot, we set the standard deviation of angles is $\sigma_{\alpha }=\frac{\pi }{18}$, $\sigma_{f}=\frac{f}{10}$. In addition, we compute $J^{T}J$ and $J^{T}r$ in Equation (12) as follows:

(11) $${\left(J^{T}J\right)}_{of}=\sum_{k\in F(o,f)}\frac{\partial {r_{of}^{k}}^{T}}{\partial \Phi_{o}}\frac{\partial r_{of}^{k}}{\partial \Phi_{f}}$$

(12) $${\left(J^{T}r\right)}_{o}=\sum_{o=1}^{n}\sum_{f\in \kappa (o)}\sum_{k\in F(o,f)}\frac{\partial {r_{of}^{k}}^{T}}{\partial \Phi_{o}}r_{of}^{k}$$

After solving the above parameters, we render panorama using multi-band blending to filter the some image edges.

## 3. Result Display and Discussion

To highlight the effectiveness of the proposed robot for multi-directional imaging sensing at a small scale, we performed experiments using different types of micro-objects using both optical microscopy and scanning electron microscopy. In order to display the texture, shape and structure of the sample more plentifully and directly, we provide different display methods for different types of samples. Namely, the panoramic image of a cylindrical sample will be built and projected into a tube, while the panoramic image of the irregularly shaped sample will be connected into a ring, blending back on itself, and then the dome image output. A description of the details of the process for dome image generation can be found in [35].

### 3.1. Multi-Directional Image Sensing Under an Optical Microscope

**Figure 5:**
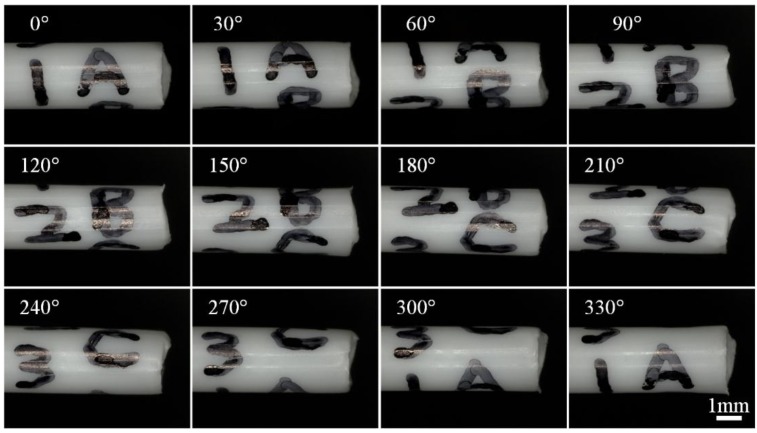
Images of the plastic tube taken from different directions under an optical microscope. Images (36) have been taken at a rate of one image every ten degrees. Twelve images have been given here as an illustration.

In this experiment, a plastic tube with diameter about 2000 µm is taken as the sample. To clearly show the difference of the images from different directions, some markers (ABC and 123) are placed on the tip of the plastic tube. First, the plastic tube is fixed on the sample stage of the robot, which is put under the lens of the microscope. Then, the sample is aligned with the rotation axis of the robot based on the alignment approach mentioned above. After that, images of the sample are taken continuously with a rotation angle of 10° every step. As the result in Figure 5 shows, this approach is able to sense all the markers (ABC and 123) on the plastic tube, which verifies the working ability of the rotation robot for multidirectional imaging. 

**Figure 6:**
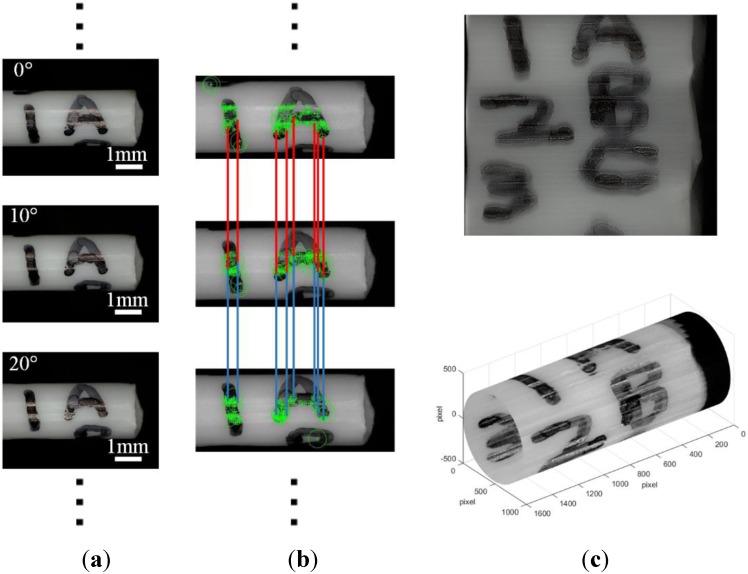
Process of generating panorama image of plastic tube. (**a**) Initial images; (**b**) feature points on images; (**c**) stitched image.

A panoramic image of the plastic tube can be constructed based on images taken at different rotation angles. The process for constructing the panoramic image is shown in Figure 6. Given a series of input images, the first step is to detect SIFT key points (Figure 6b). Here, we set all parameters as the default values in the SIFT features step, and set σ=20 in the feature matching step. Figure 6b demonstrates the correspondence of feature points in three images. According to the similarity and correspondence of feature points, RANSAC and a probabilistic verification procedure is used to find consistent image matches, *i.e.*, finding the fundamental matrix between images. Finally, connected components of image matches are detected and stitched into a panoramic image, as shown in Figure 6c. After the panoramic image is achieved, the panoramic image can also be bent, so that the bending image looks like a tube. The bending result is shown in Figure 6c. We see that the full information of the plastic tube is shown, and this information is helpful for the evaluation, optimization and selection of the sample during the nanomanipulation. 

### 3.2. Multi-Directional Image Sensing under a Scanning Electron Microscope

In addition to optical microscopy, this system is also able to image an object under SEM. Here, two micro objects, a human hair with an approximate size of 70 µm and a tungsten probe with a tip size of approximately 2 µm are taken as the samples. Firstly, each sample is put on the sample stage of the robot. Then, the robot is assembled inside the chamber of an SEM (Quanta 250, FEI Inc., Hillsboro, OR, USA). The experiment is implemented following the same procedure as that used for the OM. 

**Figure 7:**
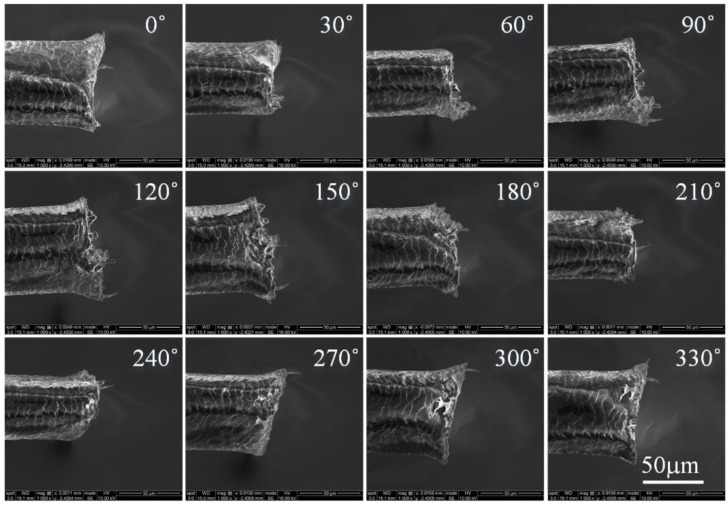
Images of human hair taken from different directions under an optical microscope. Images (36) have been taken with one image every ten degrees. Twelve images have been given here as an illustration.

**Figure 8:**
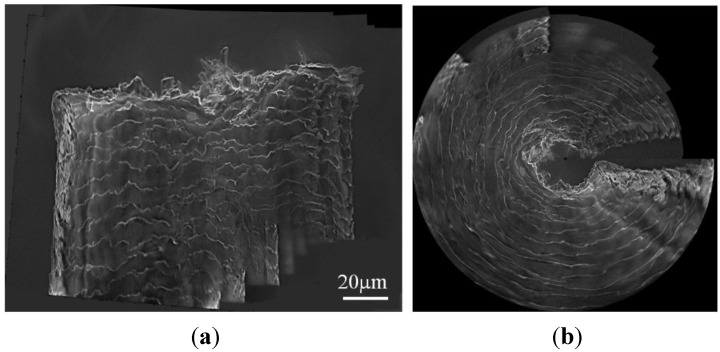
(**a**) Panoramic image of the carbon fiber and (**b**) its dome projection result.

The images of the human hair from different directions (in 10° steps) are shown in Figure 7. Here, the human hair is selected because it is not a regular cylinder under the optical microscope, and the features in images cannot be distinguished as easy as that of a plastic tube. Thus, the image stitching of the human hair is more difficult than that of plastic tube. Despite these difficulties in image stitching, our software system outputs a satisfactory result. As shown in Figure 8, Figure 8a is the panoramic image of the human hair, where we see that the field of view (FOV) of the microscope is expanded using our software system, and more detailed information, such as texture distribution and shape, can be found in the panoramic image. In order to show the panoramic image in Figure 8a more clearly, we converted the image in Figure 8 from panoramic to a dome image. From the dome image in Figure 8b, we see that the detailed structure of the human hair under the SEM provides a good view. Here, the dome image of the human hair is not closed because it is not a regular cylinder under the SEM. 

**Figure 9:**
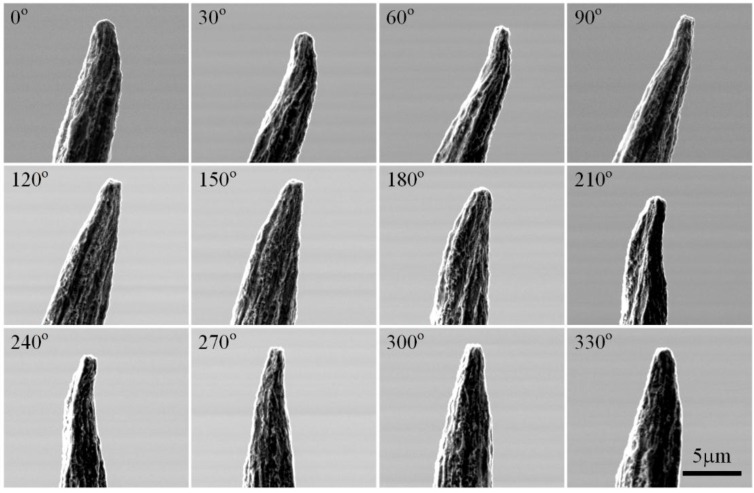
Images of the tungsten tube taken from different directions under electron scanning microscopy. Thirty six images have been taken with one image every ten degrees. Twelve images have been given here as an illustration.

**Figure 10:**
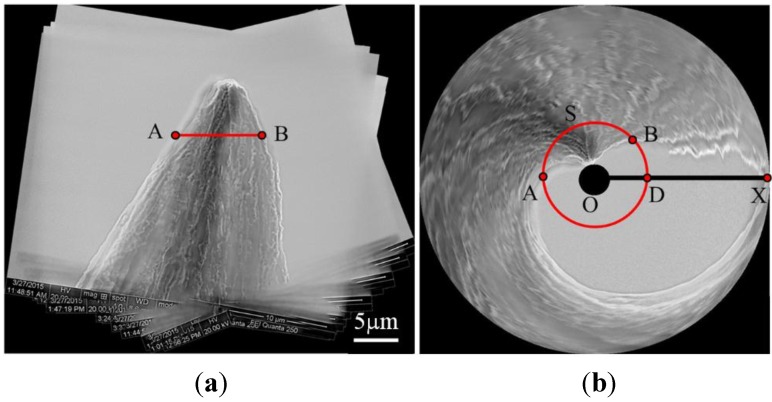
(**a**) Panorama image of the plastic tube and (**b**) its dome projection result.

In Figure 9, another sample, the tungsten probe, is selected in our test. From these images, we can clearly see the unsymmetrical structure and the uneven morphology of the tungsten probe tip. In Figure 10a, we see that the panoramic image of the plastic tube is encouraging. It means that our algorithm is more stable for matching and can handle images with varying shape. The uniformity of the observed object’s surface textures and the symmetry of the object structure can be shown in the panoramic image. 

Figure 10a shows the panoramic image of the tungsten probe. In this view, it is easy to see that the tungsten probe is not symmetrical and its surface textures are non-uniform. However, the expanded panoramic image in Figure 10a is not a very good expression of the tungsten probe, because it is not intuitive to analyze the structure of the tungsten probe using this viewpoint. Thus, we transform it into s dome image (see Figure 10b) by using the algorithm described in [35]. In this way, the surface texture and structure are displayed more plentifully, directly and strikingly. As shown in Figure 10b, the distribution of the texture from different views can be easily observed, and in that way the uneven nature of the texture can be analyzed easily. In addition, we can analyze the asymmetric structure of the sample according to the following rules. Firstly, the cylindrical sample is generally mapped onto a closed ring after the dome projection (see the result in Figure 8b as an example). Secondly, if the sample is not a cylindrical object, some blank areas will appear in the dome projection result. Thus we can estimate the structure of the sample visually based on the blank area. The more such blank areas there are, the less possibility there is for the sample to be a cylinder. This information helps us better understand the microstructure of the sample.

Taking the result in Figure 10b for example, we define one of the horizontal sections of the tungsten probe as S, which corresponds to line AB in Figure 10a and is mapped to the arc AB in Figure 10b. The intersection of the circle AB and the horizontal axis OX in Figure 10b is defined as D. Then we can estimate the structure of the tungsten probe according to the arc angle of AB and the arc angle of AD. Here, if the arc angle of AB is smaller than 2π, the horizontal section S will be not a circle, but rather it will be a non-uniform shape. If the arc angle of AB is equal to 2π, the horizontal section can be projected to a closed circle in the dome projection result, and the horizontal section is relatively smooth and similar to cylindrical sample horizontal section. And further, the bigger the arc angle of AD, the greater the tilt angle of the sample. Therefore, based on the dome projection result, we know that the tungsten probe has a large bending and it is a declining object. This information is not only helpful for the evaluation and optimization of the tungsten probe fabrication process, but also useful for the selection of the high quality end effector for nanomanipulation. Considering tungsten probes are one of the most used end effectors for nanomanipulation and nanocharacterization tasks [36,37], the structure information provides a direct path for probe modeling, thereby benefitting the precise manipulation and measurement tasks.

### 3.3. Discussion

One big challenge for image sensing at a small scale lies in the difficulty in obtaining multidirectional images since the microscope lens is usually installed at a fixed direction. This drawback not only limits our understanding of the sample, but may also lead to misleading results, since the local image information cannot reflect the overall properties of the sample. To address this issue, this paper proposes a novel robot and the corresponding software system for multidirectional sensing at a small scale.

The rotation robot is mainly made up of three nanopositioners, and thereby a high positioning accuracy can be guaranteed. In addition, the robot system has a small size and it doesn’t require the modification of the microscope imaging system when it works. Therefore, it has high usability and versatility for both OM and SEM.

To keep the sample in the FOV of the microscope during rotation is one key premise in multidirectional image sensing under a microscope. However, unlike the image sensing at a macro scale, the microscope cannot only provide a 2D local image of the sample, which makes the traditional alignment method unworkable. The proposed forward-backward alignment strategy is based on three images of the sample taken at different rotation angles. It doesn’t require any position information of the rotation axis and the sample in advance and it’s able to align the sample to the rotation axis of the robot effectively. Therefore, this approach is very convenient for the experimental preparation and implementation for both OM and SEM.

The multidirectional image sensing results under both OM and SEM prove the working efficiency of this system. They clearly show the advantages of multidirectional image sensing over the traditional method, *i.e.*, much more information about the sample can be obtained. In addition, the multidirectional imaging method is proposed to merge both OM and SEM images together. Since our method is developed based on the characteristics of the robotic system, its feature matching step is more stable and can handle images with unsymmetrical structures, varying illumination and noise. In addition, two data-display methods are provided in our software system: panoramic images and the corresponding dome projection results. The panoramic image helps analyze the distribution of the surface texture of the sample, while the blending result and the dome projection result are beneficial for the structural and shape analysis of the sample. These display methods are not only helpful for the evaluation and optimization of the tungsten probe fabrication process, but also useful for the selection of the high quality end effectors for nanomanipulation. 

The robot system integrated with the image sensing technique will provide new ways of imaging and characterization at a small scale, especially for defect detection and *in situ* characterization of samples. In the future, we will improve this system further to allow automatic 3D imaging, and build up 3D models of samples. We will also apply this system to defect detection and micromaterial characterization.

## 4. Conclusions

This paper reports a novel method for multidirectional image sensing under a microscope by developing a rotatable robot. First, a robot with endless rotation ability is designed and integrated with the microscope. Then, the micro object is aligned to the rotation axis of the robot automatically based on the proposed alignment strategy. After that, images of the sample are taken from multiple directions by rotating robot within one revolution under the microscope, and panoramic images of the sample are processed. Lastly, to demonstrate the versatility of this approach, we test various types of micro samples under both an optical microscope and scanning electron microscope. The proposed method paves a new path for microscopy imaging sensing, which could have a significant impact in many fields, especially for sample detection, manipulation and characterization at a small scale.

## Supplementary Files

Supplementary File 1
