# Supplementary material for: Multidirectional Image Sensing for Microscopy Based on a Rotatable Robot
Source: Sensors (Basel). 2015 Dec 15;15(12):31566–80. doi: 10.3390/s151229872 (PMC4721792; doi:10.3390/s151229872)
Supplement: Supplementary File 1 [file sensors-15-29872-s001.zip › sensors-15-29872-supplementary/sensors-15-29872-supplementary.pdf]

Supplementary Materials: Multidirectional Image Sensing for Microscopy Based on a Rotatable Robot

Yajing Shen, Wenfeng Wan, Lijun Zhang, Li Yong, Haojian Lu and Weili Ding

1. Alignment Experiment Demonstration

We took a tungsten needle as the sample and implemented the sample alignment based on the method proposed in Section 2.2 (main text). During this process, the to-be-aligned point at different rotation angle was recognized automatically by a Canny detection approach (Figure S1). The coordinate of the tip along the microscope image's vertical side was calculated by $x_2 + \frac{Tc - x_1 - x_2}{2}$.

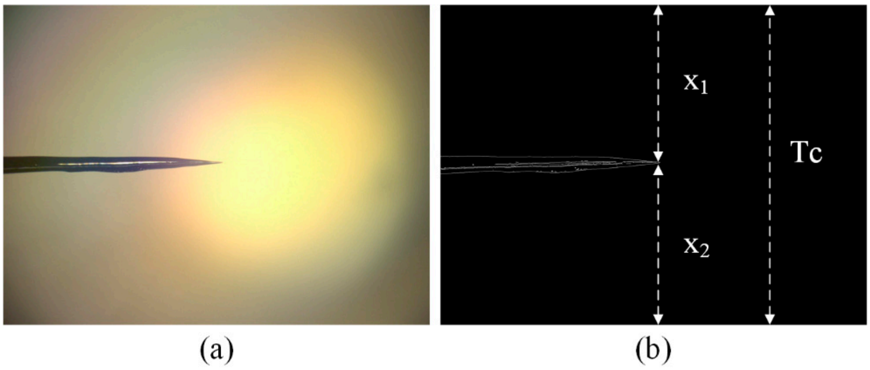

Figure S1. (**a**) The microscope image for tungsten microneedle, and (**b**) processed microscope image with Canny detection method. The coordinates of the microneedle's tip are calculated as $x_2 + \frac{Tc - x_1 - x_2}{2}$.

2. Alignment Error Justification

Figure S2a shows the microneedle's position at rotation angle −15°, 0° and 15° before alignment, and Figure S2b shows the microneedle's position at rotation angle −15°, 0° and 15° after alignment with magnification 200 and alignment angle 15°.

Before alignment, tungsten microneedle's position shift for the three images in Figure S2a is 906 μm, while after alignment, tungsten microneedle's position shift for the three images in Figure S2b has dropped largely to 8 μm. The decreasing position shift justifies the proposed method.

After that, the robot was rotated 360° at a series of rotation speed, *i.e*, 2.5 °/s, 5.0 °/s, 7.5 °/s and 10.0 °/s, respectively, and the sample was imaged dynamically from different orientation. As the results shown in the Figure S3, sample's position at different rotation angle for different speed are almost the same, which demonstrates the high repeatability of the robot.

During the 360° rotation, the maximum position shift is 84 μm. For the multidirectional imaging experiment, images of samples are taken every 30°, the maximum position shift in the 30° rotation range is 45 μm, which is much smaller than the field of view (FOV) we adopted during our multidirectional imaging experiments. Therefore, during rotation, samples would always remain in the FOV and we don't need to spend much time finding samples and adjusting samples' positions.

In conclusion, this alignment has high repeatability regardless of the rotation speed, and the alignment accuracy can meet the requirements for multidirectional imaging.

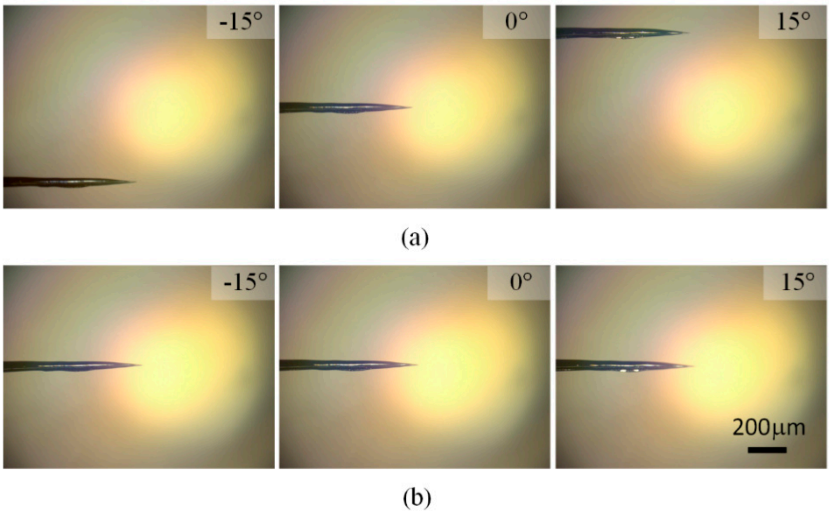

Figure S2. Microscope images at rotation angle −15°, 0° and 15° (**a**) before and (**b**) after alignment. The position shift in the three images of (a) before alignment is 906 μm, while the position shift in the three images of (b) after alignment becomes 8 μm.

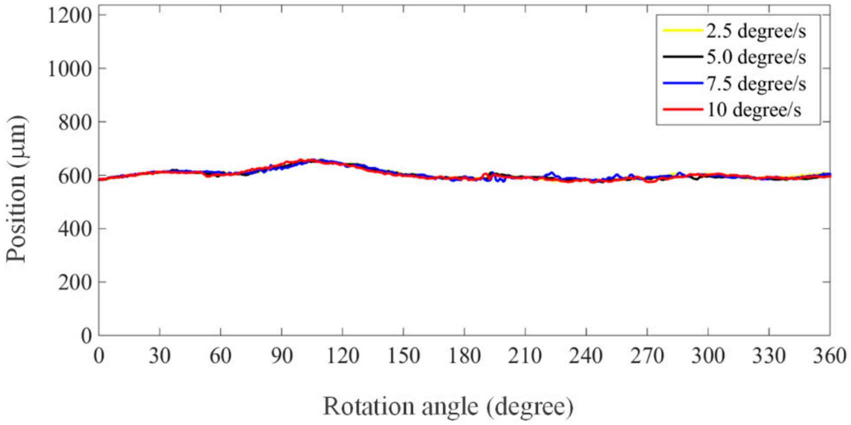

Figure S3. Sample's position at different rotation angle after alignment for various rotation speed. During the 360° rotation, the maximum position shift is 84 μm. This makes sure that samples would always keep in the field of view, and therefore avoids spending much time finding samples and adjusting samples' positions.
